# Self-Reported and Objective Sleep Measures in Stroke Survivors With Incomplete Motor Recovery at the Chronic Stage

**DOI:** 10.1177/15459683211029889

**Published:** 2021-07-01

**Authors:** Melanie K. Fleming, Tom Smejka, David Henderson Slater, Evangeline Grace Chiu, Nele Demeyere, Heidi Johansen-Berg

**Affiliations:** 1 Wellcome Centre for Integrative Neuroimaging, FMRIB, Nuffield Department of Clinical Neurosciences, 6396 University of Oxford, Oxford, UK; 2 552380 NIHR Oxford Biomedical Research Centre, UK; 3 212787 Oxford Centre for Enablement, Oxford University Hospitals NHS Foundation Trust, Oxford, UK; 4 Department of Experimental Psychology, 6396 University of Oxford, Oxford, UK

**Keywords:** actigraphy, self-report, motor impairment, sleep disruption, mood

## Abstract

*Background*. Stroke survivors commonly complain of difficulty sleeping. Poor sleep is associated with reduced quality of life and more understanding of long-term consequences of stroke on sleep is needed. *Objective.* The primary aims were to (1) compare sleep measures between chronic stroke survivors and healthy controls and (2) test for a relationship between motor impairment, time since stroke and sleep. Secondary aims were to explore mood and inactivity as potential correlates of sleep and test the correlation between self-reported and objective sleep measures. *Methods.* Cross-sectional sleep measures were obtained for 69 chronic stroke survivors (mean 65 months post-stroke, 63 years old, 24 female) and 63 healthy controls (mean 61 years old, 27 female). Self-reported sleep was assessed with the sleep condition indicator (SCI) and sleep diary ratings, objective sleep with 7-nights actigraphy and mood with the Hospital Anxiety and Depression Scale. Upper extremity motor impairment was assessed with the Fugl-Meyer assessment. *Results.* Stroke survivors had significantly poorer SCI score (*P* < .001) and higher wake after sleep onset (*P* = .005) than controls. Neither motor impairment, nor time since stroke, explained significant variance in sleep measures for the stroke group. For all participants together, greater depression was associated with poorer SCI score (*R*^2^_adj_ = .197, *P* < .001) and higher age with more fragmented sleep (*R*^2^_adj_ = .108, *P* < .001). There were weak correlations between nightly sleep ratings and actigraphy sleep measures (*r*_*s*_ = .15–.24). *Conclusions.* Sleep disturbance is present long-term after stroke. Depressive symptoms may present a modifiable factor which should be investigated alongside techniques to improve sleep in this population.

## Introduction

Many stroke survivors report a major change in their sleeping habits since having a stroke.^1^ Although there is some evidence for improvements in sleep parameters from the acute to the chronic stage of stroke,^2,3^ systematic reviews report a high prevalence of sleep disorders, such as insomnia and sleep disordered breathing, after stroke.^4,5^ Sleep disorders are reported to be more common in stroke survivors compared to normative values or to healthy control groups.^6^ Increases in estimated sleep time (per 24 hour period) from pre-stroke to post-stroke have been found to correlate negatively with the ability to engage in activities of daily living at the chronic stage^2^ and reduced quality of life has also been demonstrated for stroke survivors reporting insomnia.^7^

Although some research on sleep after stroke focuses on diagnosable sleep disorders, such as sleep disordered breathing, restless leg syndrome and primary insomnia,^5,8^ there is also research directly comparing between people with chronic stroke and age-matched controls with sleep measures as a continuum rather than categorising participants as having a sleep disorder or not. This is important, as alongside other post-stroke complications, sub-clinical sleep disruption has the potential for debilitating long-term consequences. Recently, evidence has begun to emerge that treatments, such as cognitive behavioural therapy for insomnia, may also be useful for people with sub-clinical insomnia symptoms.^9^ The studies that have analysed sleep measures as a continuum report longer sleep latency, greater wake after sleep onset (WASO) and more fragmented sleep, as well as lower subjective sleep quality for stroke survivors compared to controls.^10-13^ However, these studies typically have modest sample sizes (20–35 per group). Therefore, confirmation of these findings with a larger sample size is desirable to enable us to further understand this long-term condition.

There is some indication that sleep quality may relate to stroke outcomes, using broad measures of independence in activities of daily living^14^ or disability.^15,16^ We previously demonstrated that sleep disruption during inpatient rehabilitation from stroke and brain injury is associated with poorer motor outcomes^17^ and hypothesise that this may be, at least in part, due to impaired consolidation of motor learning underlying motor recovery.^18^ If this is the case, and if sleep disruption persists long-term, then recovery after discharge from rehabilitation may also be limited. Additionally, factors that accompany motor impairment, such as spasticity and pain, may directly affect ability to initiate and maintain sleep. Finally, limitations in movement of the upper or lower limb can affect mobility and therefore physical activity which may indirectly affect sleep. These factors may, to some extent, depend on how long the person has been living with motor impairment. Alternatively, relationships between motor impairment and sleep quality might be expected due to effects of the stroke on cortico-subcortical circuits involved both in the control of sleep and in the control of movement. For example, Gottlieb et al^19^ demonstrated that stroke survivors with poor sleep efficiency had altered brain volume in the thalamus, hippocampus and caudate in comparison with controls with normal sleep efficiency. However, to our knowledge, there are no studies at the chronic stage to consider the relationship between motor impairment and sleep quality specifically.

Mood disorders are also common post-stroke complications.^20,21^ Depression and anxiety have been found to relate to poor self-reported sleep in stroke survivors and older adults without stroke,^16,22-25^ with greater insomnia symptoms present in stroke survivors with depression or anxiety than without.^4^ However, to our knowledge, there are few studies examining whether depression and anxiety relate to objective measures of sleep in this population. Pajediene et al^26^ found a correlation between polysomnography variables reflecting poor sleep and more depressed mood (from the Hospital Anxiety and Depression Scale) in a sample of 13 acute stroke patients, though the magnitude of this correlation is not reported. In contrast, Bakken et al^27^ found no significant correlation between the Beck depression Inventory Score and actigraphy variables WASO or number of awakenings. Further research is therefore needed to elucidate whether relationships between self-report measures of mood and sleep are also seen when sleep is measured with actigraphy.

It is currently unknown whether subjective reports of sleep quality are reflective of objective measures of sleep in this population. Ouellet and Morin^28^ reported that people with traumatic brain injury subjectively reported worse sleep than control participants, but that this was not detected using polysomnography (PSG) suggesting a mismatch between objective and subjective sleep quality. In stroke survivors, 6 months post-stroke, Bakken et al^3^ report some correspondence between Pittsburgh Sleep Quality Index (PSQI) ratings and time spent asleep, but no meaningful correlations between PSQI and measures of sleep disruption from actigraphy. It is important to understand the relationship between self-reported and objective measures of sleep problems in this population, in order to best tailor interventions aimed at improving aspects of sleep quality.

The primary aims of this study were therefore to compare both objective and self-reported sleep measures between community dwelling chronic stroke survivors and age- and sex-matched healthy controls and to investigate whether variance in sleep measures in stroke survivors could be explained by variance in upper limb motor impairment or time since stroke. Additionally, we sought to explore potential correlates of sleep measures across both groups. We were particularly interested in the chronic stage of stroke, as this is the time when intensive rehabilitation efforts are likely to have completed and the long-term impact of stroke can be understood.

We hypothesised that stroke survivors would demonstrate poorer self-reported sleep, more fragmented sleep and longer time awake overnight than healthy controls. We also hypothesised that stroke survivors with a poorer motor outcome (worse upper limb impairment) would have more sleep disruption than those with good functional outcomes.

Finally, we aimed to test for differences in the agreement across subjective and objective sleep quality measures between stroke survivors and controls. We anticipated that there would be less correspondence between objective and subjective sleep measures for stroke survivors.

## Methods

### Participants

This was a prospective, cross-sectional, observational study. Potential participants were identified between July 2017 and May 2020 through the Cognitive Neuropsychology Centre at the University of Oxford, the Oxford Centre for Enablement (Oxford University Hospitals NHS Foundation Trust), online and poster advertisements, stroke support groups, word of mouth and through contacting participants from previous research studies. Inclusion criteria for stroke survivors were aged >18 years, stroke >3 months prior, self-reported difficulty using the upper limb and able and willing to provide informed consent. Exclusion criteria were neurological or psychological conditions other than stroke, diagnosed sleep disorder prior to the stroke and pre-stroke uncorrected visual impairment. Provided that participants met these inclusion criteria, individuals were not excluded on the basis of other stroke-related impairments such as dysphasia.

Healthy controls were identified through online and poster advertisements, stroke support groups and public engagement events, word of mouth and through contacting participants from previous studies who had agreed to be contacted again. Inclusion criteria for healthy controls were aged >18 years and able and willing to provide informed consent. Exclusion criteria were diagnosed sleep disorder, visual impairment or a history of neurological or psychological conditions.

We intentionally did not exclude participants for either group who reported having some difficulty sleeping, as this would have resulted in an under-representation of people with sleep problems. Equally, we ensured that people who did not think they had a sleep problem were also included, to ensure a representative sample. The study was approved by the National Research Ethics Service (11/H0605/12), and all participants provided written informed consent.

In total, 70 stroke survivors and 76 healthy controls volunteered to participate. Of these, one stroke survivor withdrew with no usable data and the data from 13 healthy controls were withdrawn as their age was outside of the range of the stroke survivors. This left a final sample of 69 stroke survivors (mean age, 63 years; range, 26–87; 24 females) and 63 controls (mean age, 61 years; range, 27–83; 27 females). Stroke survivors were on average 65 months post-stroke (range, 5–281 months). Data from 47 of the controls were reported previously.^17^

### Assessments

Our dependent variables were sleep measures obtained through self-report and using actigraphy. The self-reported sleep measures included the sleep condition indicator (SCI, max score 32)^29^ and 7-night sleep diary ratings. For the sleep diary, participants indicated what time they tried to sleep (eg turned off the light) and what time they woke each day and rated the quality of their night sleep on a 5-point scale (very good to very poor). The SCI is a validated measure to screen for insomnia symptoms and has demonstrated convergent validity with the Pittsburgh Sleep Quality Index, high internal consistency and a reliable change index of 6 points.^30,31^ Objective sleep measures were obtained with 7-night actigraphy, by placing a Motionwatch 8 (Camntech Ltd, Cambridge, UK) on each wrist for stroke survivors (with sleep measures taken from the Motionwatch of the less-affected arm) and the non-dominant wrist for controls. The actigraph can be used to predict when the body is in periods of sleep under the assumption of the body being motionless during deep sleep, in comparison to wake. Actigraphy is an acceptably valid and reliable method for measuring sleep patterns and symptoms of insomnia in the home environment.^32^

Potential explanatory variables included demographic variables (age, sex and time since stroke), mood, motor impairment and inactivity. Mood was assessed for both groups using the hospital anxiety and depression scale (HADS, max score 21 per subscale). For stroke survivors, upper limb motor impairment was assessed using the upper extremity portion of the Fugl-Meyer assessment (UE-FM, max score 66).^33^ Inactivity was determined from actigraphy, as the number of minutes spent sedentary over each 24-hour period.

### Data Analysis

The Motionwatch 8 contains an accelerometer and motion is converted into an activity count. Data were averaged by the monitor into 30 s epochs. Sleep measures were extracted using the custom software, Motionware (Camntech Ltd), using a threshold of 20 (high sensitivity). Since it was not always possible to rely on the event marker in this population, the time that participants tried to sleep (eg turned off the light) and got up was taken from the sleep diary and adjusted based on the apparent movement and time of the event marker if present. Sleep measures included assumed sleep duration (ie the time spent in bed with the intention of sleeping, minutes), actual sleep duration (according to the epoch-by-epoch wake/sleep categorisation, minutes), WASO (minutes) and the fragmentation index (the sum of the total time categorised as mobile, expressed as a percentage of the assumed sleep, and the number of immobile bouts which were ≤1 min in length, expressed as a percentage of the total immobile bouts). We also extracted sedentary time (minutes) over each 24-hour period and average movement per 24-hour period (motion-units) for each arm of the stroke group to investigate relative differences in movement for the impaired vs less-affected arm.

Data were analysed using SPSS 25 (IBM inc) and GraphPad Prism 8 (GraphPad software LLC). Differences in sleep measures between stroke survivors and controls were tested using Mann–Whitney U tests, or t-tests if appropriate, following assessment of normality. An adjusted significance value of *P* < .006 was used to correct for multiple comparisons. One-sided tests were used for variables shown previously to be worse for stroke survivors compared with controls (WASO, fragmentation index and self-reported sleep quality). The sleep diary ratings (very poor, poor, fair, good and very good) were dummy-coded (where 1 = very good and 5 = very poor) for analysis.

To specifically address the question of whether motor impairment is related to SCI score or sleep disruption from actigraphy, we conducted 3 stepwise regression models for the stroke group only, with independent variables of UE-FM score and time since stroke and dependent variables of SCI score, WASO and fragmentation index. An adjusted significance of *P* < .017 was used to account for 3 regression models.

In order to also explore whether symptoms of depression or anxiety, or inactivity related to sleep in both stroke survivors and controls, we conducted additional stepwise regression models for the 3 dependent variables (SCI score, WASO and fragmentation index) with independent variables of group (stroke/control), age, anxiety subscale of HADS score, depression subscale of HADS score, inactivity (sedentary time) and sex (male, female). An adjusted significance of *P* < .017 was used to account for 3 regression models. Outliers were detected using GraphPad Prism 8 ROUT tool and visual inspection of the data. Pairwise deletion was utilised to enable associations between variables to be calculated in the case of missing data in one variable, and multi-collinearity was checked using the variance inflation factor (all <2 indicating acceptably weak multi-collinearity).

Coherence between objective and self-reported measures of sleep quality was assessed using the dummy-coded sleep diary ratings (where 1 = very good and 5 = very poor) and correlated with sleep fragmentation and WASO values for the corresponding night. Spearman correlation coefficients were calculated for each group (stroke and controls). The strength of the correlations was compared across groups using Fisher’s *r*_*s*_ to *Z* test.

## Results

### Differences Between Stroke Survivors and Controls

All participants fully completed the questionnaires. Five stroke survivors and one control had no actigraphy data due to removal of the actigraphy monitor with fewer than 3 nights assessed (n = 3), unreadable data (n = 2) or a lost actigraphy monitor (n = 1). Compliance was otherwise good, with 90% of the remaining participants wearing the actigraphy monitor for the full week. There were no differences between groups for demographic variables age (*t* (130) = .860, *P* = .392) or sex (χ^2^ = .906, *P* = .341).

Data comparing stroke survivors and controls are shown in Table 1. Based on actigraphy data, stroke survivors had significantly longer assumed sleep duration (*t* (109.5) = 3.629, *P* < .001) and actual sleep duration than controls (*U* = 1411, *P* = .005), indicating that stroke survivors spent more time in bed with the intention of sleeping and actually asleep than controls. As expected, the stroke group had significantly poorer SCI score (*U* = 1434, *P* < .001) and sleep diary ratings tended to be poorer (*U* = 1598, *P* = .009) than for controls, which was not significant with the Bonferroni correction. An SCI score of 16 or less indicates probable insomnia and as such 35% of the stroke group would meet the criteria for a probable insomnia disorder in comparison to 11% of the control group (χ^2^ = 10.269, P=.001). Actigraphy data indicated that the stroke group spent significantly more time awake overnight (WASO: *U* = 1480, *P* = .005). Sleep fragmentation index tended to be higher (more disrupted) but was not significant with the Bonferroni correction (*U* = 1514, *P* = .010).

**Table 1.** Characteristics and sleep variables for each group.

		Stroke (n = 69)	Controls (*n* = 63)	*P*	Cohen’s *d*
Age – years					
	*Mean (range)*	63 (26–87)	61 (27–83)	.392	—
Sex					
	*Male:Female*	45:24	36:27	.341	—
SCI					
	*Median (range)*	21 (5–32)	27 (0–32)	<.001	0.6
Sleep diary rating					
	*Median (range)*	3 (1–4)	2 (1–4)	.009	0.4
HADS-anxiety					
	*Median (range)*	5 (0–15)	4 (0–12)	.016	0.4
HADS-depression					
	*Median (range)*	5 (0–14)	2 (0–10)	<.001	1.2
Assumed sleep – minutes					
	*Mean (range)*	505 (311–811)	465 (340–570)	<.001	0.6
Actual sleep – minutes					
	*Median (range)*	435 (238–753)	405 (305–512)	.005	0.4
WASO – minutes					
	*Median (range)*	61 (14–158)	49 (23–111)	.007	0.4
Fragmentation					
	*Median (range)*	33 (3–74)	26 (12–62)	.010	0.4
Sedentary time – minutes					
	*Mean (range)*	917 (641–1185)	858 (596–1141)	.008	0.5
Upper extremity Fugl-Meyer score					
	*Median (range)*	36 (5–66)	N/A	—	—

Abbreviations: HADS, hospital anxiety and depression scale; SCI, sleep condition indicator; WASO, wake after sleep onset. Assumed sleep, Actual sleep, WASO and Fragmentation and Sedentary time are obtained from actigraphy using custom software (Motionware).

### Exploring Factors Affecting Sleep Quality

**Figure 1.**
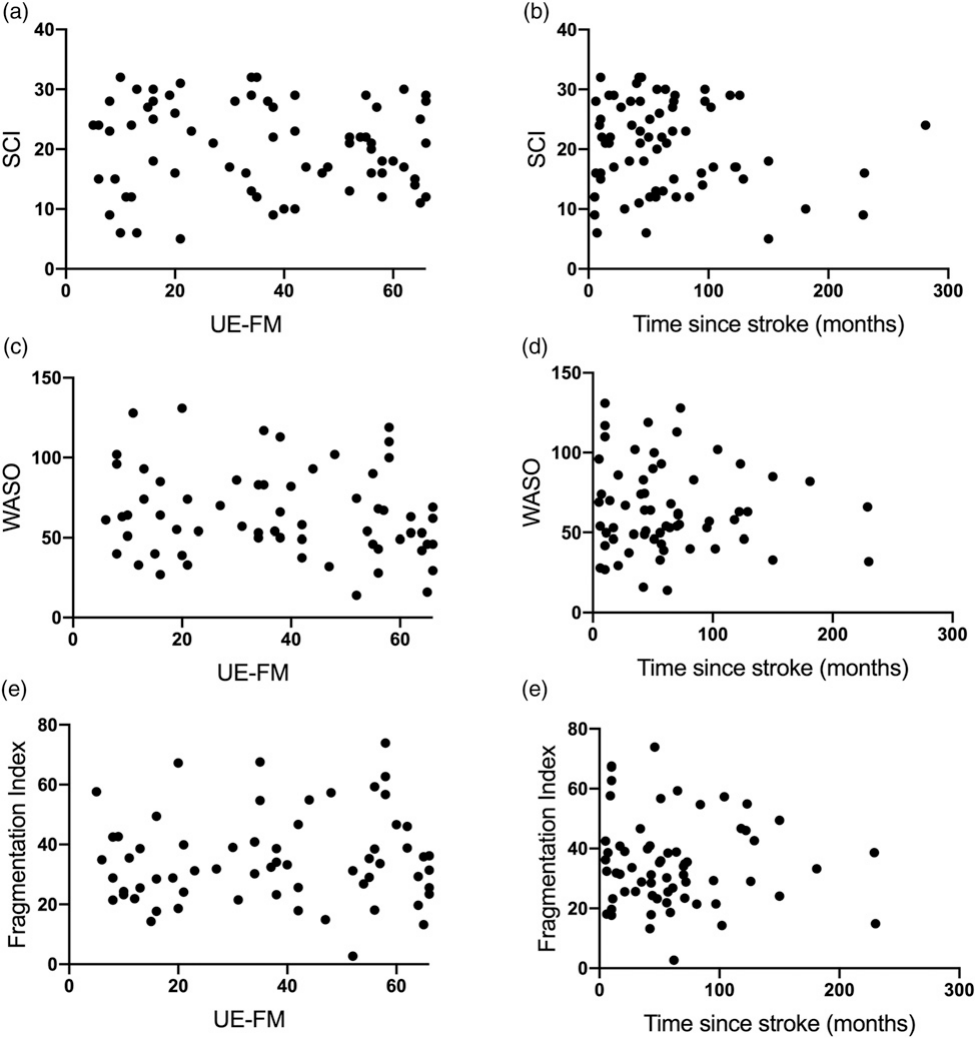
Neither motor impairment nor time since stroke explains variance in sleep measures for the stroke group. A–C: Higher upper extremity Fugl-Meyer (UE-FM) indicates less motor impairment. SCI (A, D) = sleep condition indicator: higher values indicate better perceived sleep. WASO (B, E) = wake after sleep onset. Higher WASO or sleep fragmentation index (C, F) indicates more disrupted sleep.

As we expected for stroke survivors with motor impairment, the affected arm moved less per 24 hour period than the ‘unaffected’ arm (median affected: 20 motion-units, unaffected: 56 motion-units, *W* = 1557, *P* = .001). We sought to determine whether sleep quality was related to upper limb impairment or time since stroke. However, neither UE-FM nor time since stroke was found to explain variance in SCI score, WASO or fragmentation index (all *P* > .05; Figure 1).

**Figure 2.**
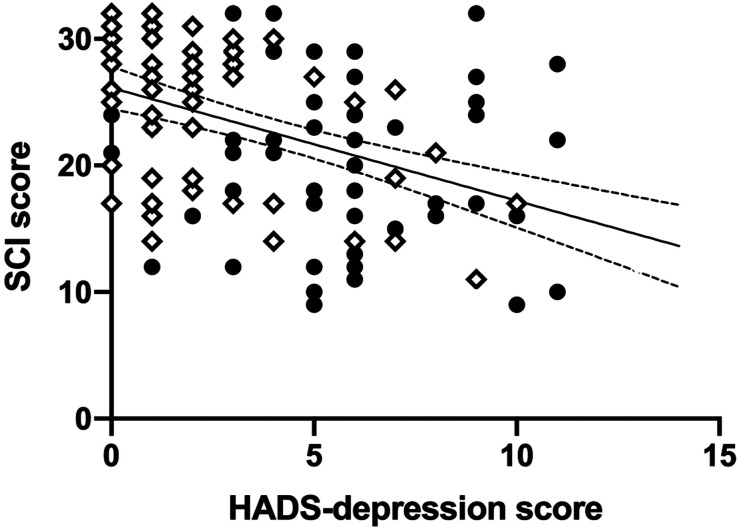
Self-reported depressive symptoms score explains 20% of the variance in self-reported sleep with all participants together. SCI, sleep condition indicator; higher values indicate better perceived sleep. HADS, hospital anxiety and depression scale, depression subscale score; higher values indicate more depressive symptoms. Filled circles, stroke group, open diamonds = control group. Simple linear regression line shown with 95% confidence bands.

We also sought to explore whether SCI score, WASO or fragmentation index in the entire sample could be explained by mood (HADS-anxiety and HADS-depression) or inactivity (sedentary time), in addition to demographic variables (group (stroke/controls), sex or age). For SCI score, HADS-depression score was found to explain 20% of the variance (*R*^2^_adj_ = .197, *F*_1,120_ = 30.659, *P* < .001) such that people with more depressive symptoms had more self-reported symptoms suggestive of insomnia (Figure 2). Adding HADS-anxiety to the model increased the variance explained to 22.6%, but this increase was not significant with Bonferroni correction (Δ*R*^2^ = .032, *F*_1,119_ = 4.994, *P* = .027). No other variables were found to contribute significantly to the model. This suggests that this relationship was not specific to the stroke group.

For WASO, group was found to explain a small, but significant proportion of the variance (*R*^2^_adj_ = .047, *F*_1,120_ = 5.89, *P* = .017), indicating that the control group had lower WASO as reported in Table 1. However, no other variables contributed to the model.

**Figure 3.**
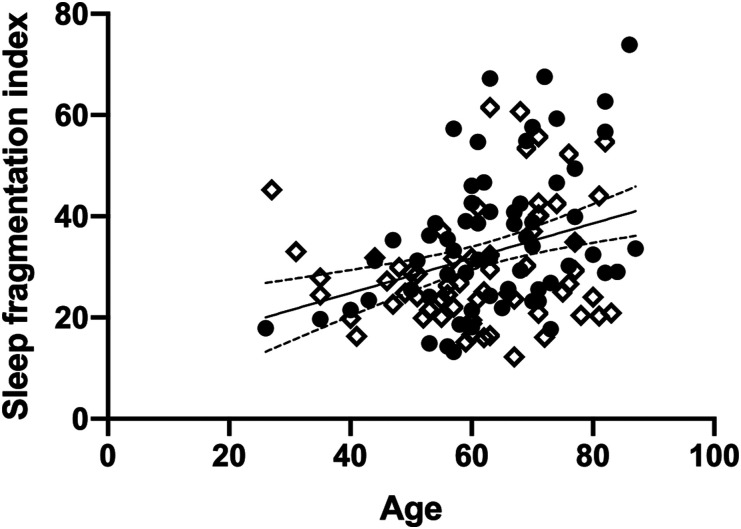
Age explains 11% of the variance in sleep fragmentation index, whereby higher age (years) is associated with more disrupted sleep. There was a tendency for this relationship to depend on group (stroke/controls). Filled circles = stroke group, open diamonds = control group. Simple linear regression line shown with 95% confidence bands.

For sleep fragmentation index, age was found to explain 11% of the variance (*R*^2^_adj_ = .108, *F*_1,120_ = 14.60, *P* < .001), such that higher age was associated with more fragmented sleep (Figure 3). Adding group increased the variance explained to 13.2%, but this change was not significant with the Bonferroni correction (ΔR^2^ = .038, *F*_1,119_ = 5.23, *P* = .024). This suggests that there is a tendency for more disrupted sleep in particular when older age is combined with history of stroke.

### Correlation Between Objective and Subjective Sleep Quality

**Figure 4.**
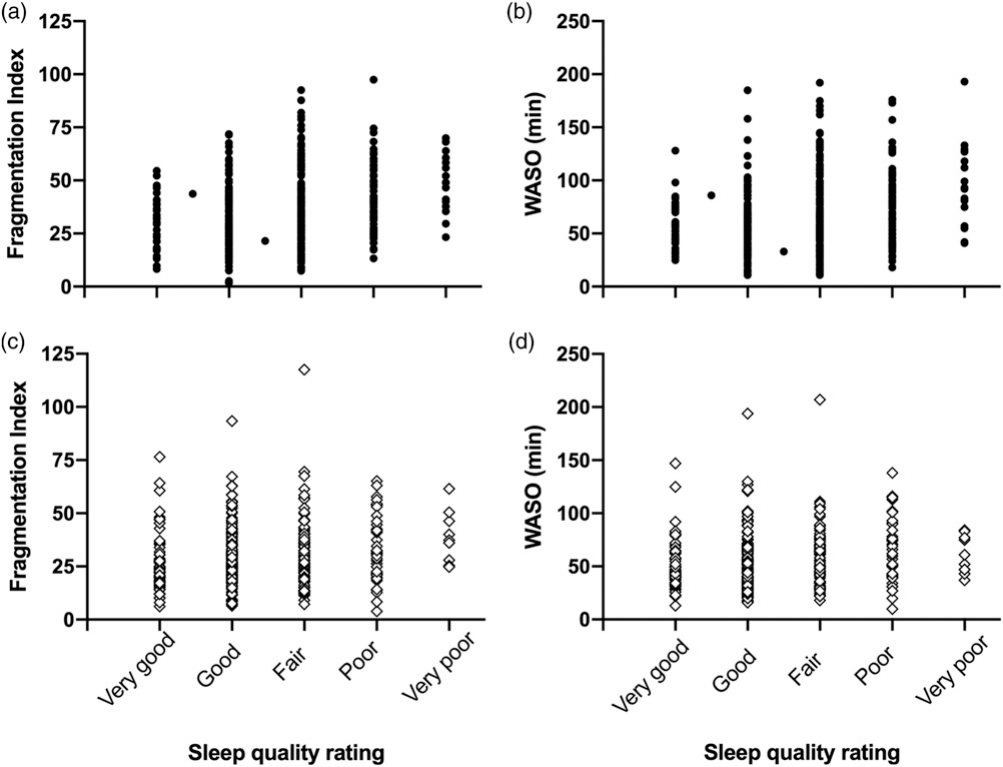
Significant correlation between subjective nightly sleep quality rating and objective sleep measures, fragmentation index (A, C) and wake after sleep onset (WASO: B, D). Top = stroke survivors (filled circles), bottom = controls (open diamonds). Sleep quality was dummy-coded (where 1 = very good and 5 = very poor) for correlation analyses. Higher fragmentation index or WASO indicates more disrupted sleep.

Table 2 and Figure 4 show that both stroke survivors and healthy controls demonstrated a significant correlation between nightly sleep quality rating (1–5; where 1 = very good and 5 = very poor) and fragmentation index/WASO (where higher values indicate poorer sleep) indicating that better self-reported sleep quality was generally associated with less sleep disruption. However, in all cases, the strength of this correlation was low, suggesting poor correspondence, which did not differ significantly between groups (Fisher’s *r*_*s*_ to *Z*: sleep fragmentation: *P* = .081, WASO: *P* = .338).

**Table 2.** Correlations between nightly sleep ratings and objective sleep variables.

	Fragmentation index			WASO		
	*r*_*s*_	95% CI	*P*	*r*_*s*_	95% CI	*P*
Stroke	.24	.14–.33	<.001	.20	.10–.29	<.001
Controls	.15	.05–.25	.003	.17	.07–.27	<.001

*r*_*s*_, Spearman correlation, CI, confidence interval for *r*_*s*_. WASO, wake after sleep onset. Positive correlations indicate that poorer diary ratings are associated with worse objective sleep measures.

## Discussion

This study confirms previous findings that community dwelling, chronic stroke survivors experience worse self-reported sleep than people who have not had a stroke and spend more time awake overnight. This is in a context of more time spent in bed trying to sleep and longer overall sleep duration in stroke survivors. Based on total SCI scores,^29^ 35% of the stroke group would meet the criteria for a probable insomnia disorder, compared with 11% of the control group. However, neither SCI score nor actigraphy measures of sleep disruption were related to severity of motor impairment, nor time since stroke in this sample of chronic stroke survivors. Rather, for both stroke survivors and controls, those with more depressive symptoms reported poorer sleep, and older age was associated with more fragmented sleep.

We were surprised that neither motor impairment nor time since stroke was found to relate to subjective or objective sleep measures. Although no previous studies have specifically examined the relationship between motor impairment and sleep disruption measures at the chronic stage of stroke, generally it has been reported that people with more severe stroke self-report worse sleep quality.^14,15,34^ The lack of findings here suggests that the relationships found previously may be influenced by other factors that contribute to stroke severity scores, over and above motor impairment. Information on stroke severity (eg clinical stroke severity scores or lesion volumes) was not available or collected in the current study and therefore we are unable to test this possibility directly. However, we previously reported that sleep disruption measured with actigraphy throughout inpatient rehabilitation at the sub-acute stage of brain injury explained variance in motor outcomes, whereby those with poorer sleep had worse outcomes.^17^ This was particularly evident for measures that combined the upper and the lower limb, or were focused on mobility. Unfortunately, due to logistical space and safety constraints, we were unable to assess lower limb motor impairment in the current study, so it still remains to be seen whether lower limb motor measures would relate to sleep quality at this chronic stage of recovery. The neuroanatomical circuitry involved in sleep disturbance after stroke is not well understood; post-stroke sleep–wake dysfunction may depend on the location and extent of the lesion, and/or subsequent changes in brain volume or connectivity between regions involved in the sleep–wake cycle.^19,35^ Unfortunately, in the present study, we did not have access to original brain imaging, and longitudinal studies are needed evaluating the association between multivariate brain imaging measures and sleep changes over time after stroke.

The time since stroke varied considerably across participants in the current study but did not relate to SCI score or sleep disruption measures from actigraphy. Previous studies demonstrate some, albeit limited, improvements in sleep continuity from the acute stage to 6-month post-stroke,^2,3^ but our results suggest that sleep problems are maintained to some extent even years after stroke. However, this dataset did not include anyone in the acute or early sub-acute stage of stroke (minimum time since stroke was 5 months) and more longitudinal data are needed to enable conclusions about changes in sleep problems over time and to explore whether early intervention could impact on long-term sleep outcomes in this population.

It has been consistently shown that there is a bidirectional relationship between perceived sleep quality and mood, whereby better sleep one night is associated with more positive mood the next day, and daytime positive mood is associated with better self-reported sleep quality that night.^36^ Our results are consistent with this finding, indicating that irrespective of whether someone has had a stroke or not, higher ratings of depressive symptoms are associated with worse SCI score. Studies have shown that people with insomnia are at higher risk of developing depression than those without sleep difficulties^37^ and a higher rate of insomnia symptoms in the stroke population may therefore predispose them to depression. In the current study, HADS-depression score was found to be significantly higher for the stroke group than the control group, and previous studies report approximately a quarter of stroke survivors to experience depression and anxiety in the first years after stroke.^20,21^ However, HADS-depression was not found to explain significant variance in objective sleep disruption measures, and there was poor correspondence between self-reported sleep quality ratings and objective sleep measures. Therefore, it must be considered whether depressive symptoms lead participants to answer questions more negatively than their true sleep experience. Nevertheless, depression represents a potentially modifiable factor that could be targeted alongside insomnia in this population. For example, digital cognitive behavioural therapy for insomnia has shown efficacy in improving co-morbid mood disorders,^38^ which warrants investigation in stroke survivors.

Our findings that older age was associated with more sleep fragmentation are consistent with previous research.^39^ The tendency for this to be dependent on group leads us to speculate that stroke may exacerbate the age-related decline in sleep quality, potentially through brain volume changes. Atrophy of the frontal lobe has been suggested as a cause of age-related sleep dysfunction,^40,41^ and reduced subcortical regional volume has been detected in stroke survivors with poor sleep efficiency compared to good sleepers.^19^ We included sedentary time as a factor in the current study as previous studies have shown stroke survivors to be less active than controls,^42^ age is associated with reductions in activity^43^ and regular physical activity is associated with better sleep quality in the general population.^44^ However, in the present study, sedentary time was not found to be a factor explaining significant variance in SCI score or sleep disruption from actigraphy.

Although there was a statistically significant correlation between subjective ratings of sleep quality each night and objective measures WASO and fragmentation index for both groups, these were weak correlations. This suggests that the ability of older adults and stroke survivors to accurately rate their sleep quality is limited, consistent with findings in other disorders.^45^ This further highlights the importance of considering both objective and self-reported measures in studies investigating sleep in these populations. Additionally, it will be important for studies in the future to test interventions aiming to change the way that people think about their sleep, in addition to attempting to change sleep architecture itself.

### Limitations

There are clear limitations that should be considered for future studies. Firstly, the majority of the stroke survivors in the current study were many years post-stroke. This was an advantage in allowing us to consider the long-term consequences of stroke, and there was no clear relationship between time since stroke and sleep measures. Nevertheless, it would potentially have been helpful to have also included patients who were more recently discharged from hospital/rehabilitation. Secondly, our inability to record lower limb motor impairment measures means that we are unable to rule out the possibility that a combined upper and lower limb measure would relate to sleep disruption. We also did not seek to assess the potential confounds of fatigue on the relationships explored here. There is some indication that post-stroke fatigue is associated with reduced motor cortex excitability,^46^ impaired mobility^47^ and with sleep disorders,^48^ and fatigue could therefore mediate the relationship between motor impairment and sleep parameters. Fatigue is also associated with depressive symptoms,^48^ even in participants without a diagnosis of depression.^47^

Similarly, we did not have any measures of cognitive impairment, severity or type of stroke which could also have influenced sleep and self-report measures.

## Conclusions/Implications

Sleep disturbance is present long-term after stroke, but does not appear to be related to upper limb motor impairment. Depression may present a modifiable factor which could influence perceived sleep quality and should be investigated in future studies seeking to test whether sleep can be improved in this population.
